# In search of the next super models

**DOI:** 10.15252/emmm.201911502

**Published:** 2019-11-18

**Authors:** Alexander Goedel, Niels Grote Beverborg, Makoto Sahara, Kenneth R Chien

**Affiliations:** ^1^ Department of Cell and Molecular Biology Karolinska Institutet Stockholm Sweden; ^2^ Department of Surgery Yale University School of Medicine New Haven CN USA; ^3^ Department of Medicine Huddinge Karolinska Institutet Stockholm Sweden

**Keywords:** Cardiovascular System, Genetics, Gene Therapy & Genetic Disease, Regenerative Medicine

## Abstract

The advent of pluripotent stem cell biology and facile genetic manipulation via CRISPR technology has ushered in a new era of human disease models for drug discovery and development. While these precision “super models” hold great promise for tailoring personalized therapy, their full potential and *in vivo* validation have remained elusive.

## Graphical abstract

Pluripotent stem cells and CRISPR facile technology have ushered in a new era of human disease models for drug discovery and development. Goedel, Chien and colleagues discuss these “super models” for precision medicine in the context of the work from Prondzynski *et al*.

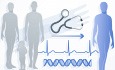

In this issue of *EMBO Molecular Medicine*, Prondzynski *et al* (2019) take a step toward the next generation of “super models” by combining deep‐clinical phenotyping, genomic sequencing, and hiPSC‐based disease modeling in an elegant study highlighting how the combination of these tools could be utilized for precision medicine. They investigated the genetic background of a family with a familial form of hypertrophic cardiomyopathy (HCM) using next‐generation sequencing and identified a novel mutation in the gene *ACTN2*. The protein encoded by the gene is α‐actinin 2 which is part of the sarcomeres, the force generating apparatus of the heart muscle.

Since human cardiac tissue for further functional analysis of the pathomechanism of this mutation is difficult to obtain, human induced pluripotent stem cells (hiPSCs) of one of the affected family members were generated. Through detailed *in vitro* analysis of patient‐specific hiPSC‐derived cardiomyocytes as well as isogenic control cells and computer modeling, they uncovered that the mutated form of the protein has an aberrant physical interaction with the L‐type calcium channel, resulting in altered calcium signaling of the cardiomyocytes. This ultimately leads to a prolongation of the action potential duration. Interestingly, the clinical investigations of the patients had revealed a prolonged QT interval in the surface ECG, which corresponds to a prolonged action potential duration on the cellular level. Applying diltiazem, which is a L‐type calcium channel antagonist clinically used as an antihypertensive drug, to patient‐specific cardiomyocytes *in vitro* led to a shortening of the action potential. Transferring this knowledge from bench back to the bedside, two patients from the affected family that showed substantial prolongations of the QT time in their surface ECGs were prescribed diltiazem, which led to a significant reduction in their QT times.

This study makes a strong case for continued rigorous and ingenious efforts to unlock the full potential of pluripotent stem cell models by moving toward 3‐D tissue models, coupling state of the art precision genetically engineering with novel tissue engineering platforms. At the same time, it should be noted that the achieved clinical benefit of shortening of the QT time in these patients could be considered relatively modest, since the arrhythmogenic burden in the affected family is low, as noted by the authors. Whether this treatment also affects hallmarks of HCM like cardiomyocyte hypertrophy and fibrotic tissue formation has not been assessed in this report and would require extensive follow‐up. Nevertheless, the combination of careful clinical phenotyping, genetic analysis, and advanced *in vitro* disease modeling appears to be a promising approach for precision therapy (Fig 1).

**Figure 1:**
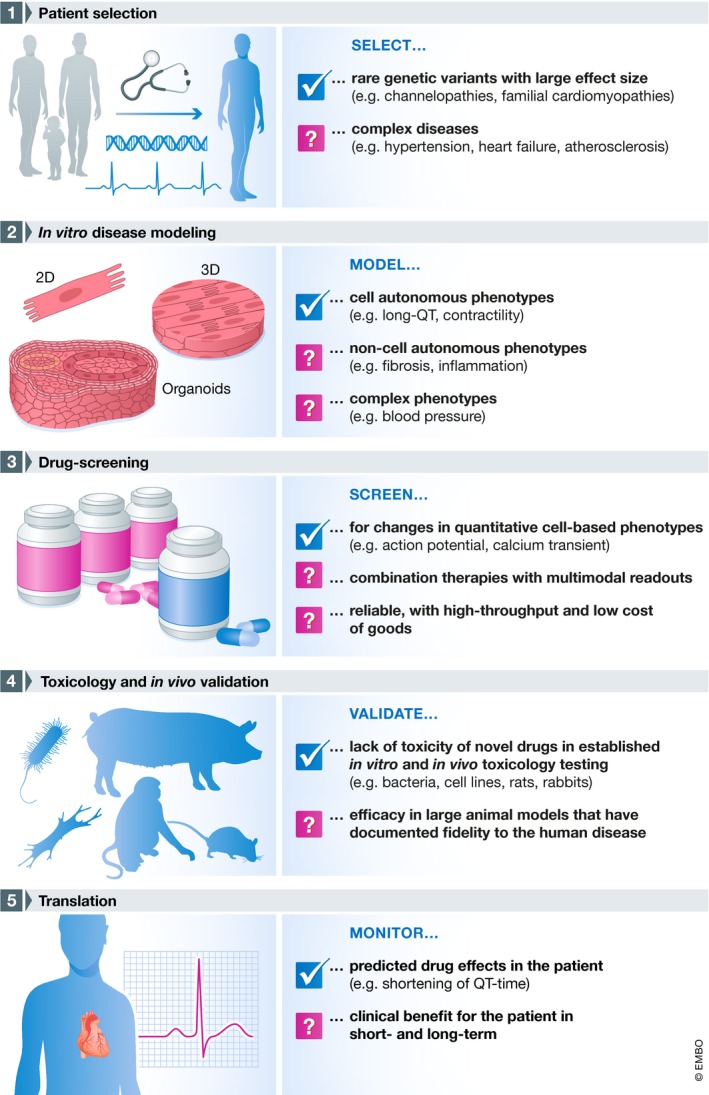
Concept, goals, and challenges of precision medicine Schematic outline of the workflow for precision medicine (left side) and the associated goals and challenges (right side).

The tortuous path from bedside to bench and back to the bedside is long and needs substantial effort and resources. In settings where individuals have a high disease burden despite optimal standard therapy, such an investment seems justified. Recently, Kim *et al* reported about a targeted oligonucleotide therapy for a girl with a severe, rare genetic disease (Batten's disease). This therapy was developed using an *in vitro* assay with patient‐specific fibroblasts and is only applicable for this specific patient (Kim *et al*, 2019). In other cases, the same disease‐causing mutation is found in large cohorts of patients. For example, the mutation R14del in the gene *PLN* affects thousands of patients posing them at high risk for developing heart failure, malignant ventricular arrhythmias, and increased mortality. The phenotype appears more malignant than other forms of dilated or arrhythmogenic cardiomyopathies, and a proven effective treatment is lacking. It has already been shown that this phenotype can be modeled with hiPSC‐derived cardiomyocytes *in vitro* (Karakikes *et al*, 2015) and efforts to identify a tailored therapy are currently ongoing. This specific situation is located on the intersection between precision medicine and “classical” drug screening, since it uses patient‐specific material for a disease model, but the tailored therapy that comes out of a screening could be applied to thousands of patients.

Considering that the technology behind hiPSCs was discovered only about a decade ago, the progress made so far is substantial. However, hiPSC‐derived cells are immature and resemble more their embryonic counterparts than mature adult cells, which render a direct translation of the *in vitro* findings to the clinical setting difficult. Moreover, variability between different cell lines and laboratories remains a major challenge. Improvements in cell culture conditions and 3‐D culture systems have led to a further maturation of hiPSC‐derived cardiomyocytes (Tu *et al*, 2018). Novel optical readout tools allow to study complex arrhythmias in *in vitro*‐derived 3‐D tissues (Kawatou *et al*, 2017) and enable simultaneous recordings of different features of cardiomyocyte biology, that increases the fidelity of the acquired data (van Meer *et al*, 2019).

Some of the phenotypes caused by genetic mutations only become evident in the crosstalk between different cells and cell types. The self‐organizing capacity of embryonic stem cells can be utilized to form organ‐like structures *in vitro* (organoids) that contain various cell types (Lancaster & Huch, 2019). Developing cardiac organoids from pluripotent stem cells has been challenging and, despite some success (Mills *et al*, 2019), has not reached the same level of fidelity as for brain, gut, or kidney. Alternative approaches combine *in vitro*‐derived fibroblasts, endothelial cells, and cardiomyocytes into microtissues, that show improved function as compared to a classical 2‐D culture and allow to study crosstalk between these cell types (Savoji *et al*, 2019). First efforts have been made to combine these *in vitro*‐derived tissues with organ‐chip technology, which enables more detailed readouts, increased throughput, and, in combination with other “organs”, could even mimic a multi‐organ response (Ronaldson‐Bouchard & Vunjak‐Novakovic, 2018).

Despite these improvements, key aspects of cardiomyocyte biology remain distinct between hiPSC‐derived cells and their native counterparts. Moreover, organ‐wide pathologies such as fibrosis of cardiac tissue, changes in wall stress, or dilation of the left ventricle are difficult to model with the technology currently available. This limitation becomes also evident in the study of Prondzynski *et al* They were able to predict effects of diltiazem on action potential morphology, which can be investigated in hiPSC‐derived cardiomyocytes fairly easily, but not on fibrosis or heart failure, since these aspects of the disease are not reflected well in the *in vitro* model.

As with any new evolving technology, there are always good news and bad news. For cardiovascular scientists and physicians, the good news is that the disease is in the dish. For cardiac patients, the bad news is that the disease is in the dish, and there has yet to be full validation that a novel drug can be identified in pluripotent stem cell models and be fully validated by FDA approval after carefully controlled clinical trials. As such, the search for the next humanized cardiovascular “super models” continues. As they say, stay tuned.

## Conflict of interest

A.G. and M.S. declare that they have no conflict of interest. N.G.B. receives a research grant from the PLN Patient Foundation. K.R.C. is a scientific founder and equity holder in Moderna Therapeutics and Procella Therapeutics, and chair of the External Science Panel for AstraZeneca.
